# Modeling Thermal Developmental Trajectories and Thermal Requirements of the Ladybird *Stethorus gilvifrons*

**DOI:** 10.3390/insects14010011

**Published:** 2022-12-22

**Authors:** Maryam Jafari, Shila Goldasteh, Hossein Ranjbar Aghdam, Abbas Ali Zamani, Ebrahim Soleyman-Nejadian, Peter Schausberger

**Affiliations:** 1 Department of Entomology, College of Agriculture, Arak Branch, Islamic Azad University, Arak 6134937333, Iran; shilagoldasteh@yahoo.com (S.G.); soley322@rocketmail.com (E.S.-N.); 2 Iranian Research Institute of Plant Protection, Agricultural Research, Education and Extension Organization, Tehran 1475744741, Iran; hrap1388@gmail.com; 3 Department of Plant Protection, College of Agriculture, Razi University, Kermanshah 6718773654, Iran; azamani@razi.ac.ir; 4 Department of Behavioral and Cognitive Biology, University of Vienna, 1030 Vienna, Austria

**Keywords:** *Stethorus gilvifrons*, development time, *Tetranychus urticae*, temperature thresholds, linear and non-linear model, thermal constant

## Abstract

**Simple Summary:**

The ladybird *Stethorus gilvifrons* is an important natural enemy of herbivorous two-spotted spider mites, *Tetranychus urticae*. Here, we modeled the effect of temperature, which is the most significant abiotic environmental factor, on the development and thermal thresholds of *S. gilvifrons*. Thermal parameters and developmental trajectories were estimated by fitting two linear and 20 non-linear models to experimental data measured at seven temperature regimes ranging from 15 to 38 °C. Our findings demonstrate that *S. gilvifrons* is able to complete development at a broad range of temperatures and is well-adapted to temperatures occurring in temperate, Mediterranean, and subtropical climates. The thermal development models presented here represent a significant step towards an in-depth evaluation of *S. gilvifrons* as a biological control agent of *T. urticae* under different temperature regimes. The models can be used to predict the phenology of this predator, to forecast its population dynamics in the field, and to optimize mass-rearing efforts.

**Abstract:**

The development rate of the predatory ladybird, *Stethorus gilvifrons* (Mulsant), fed on *Tetranychus urticae* Koch, was determined at 15, 20, 25, 27, 30, 34, and 38 °C. The total development time from egg to adult emergence for females was estimated to be 61.4, 31.6, 14.4, 13.3, 12.5, and 11.7 days, respectively. The development time decreased with increasing temperature from 15 to 34 °C, but all eggs failed to hatch at 38 °C. The lower temperature threshold (*T*_0_) for the entire development period and the thermal constant (*K*) for female *S. gilvifrons* were estimated to be 11.64 °C and 194.50 degree-days (DD) using the common linear model, and 11.96 °C and 187.87 DD using the Ikemoto and Takai model, respectively. Data were fitted to 20 non-linear development rate models and the thermal thresholds (*T_min_* and *T_max_*) and optimal temperature (*T_opt_*) were estimated. Among non-linear models, the Briere-2 and Ikemoto and Takai linear model provided adequate descriptions of the temperature-dependent development of *S. gilvifrons*. The upper-temperature threshold was estimated to be about 44 °C using the Logan-10 non-linear model. The estimated thermal development characteristics can be used to predict the occurrence and the population dynamics, as well as to improve the mass rearing and release, of *S. gilvifrons* for the biological control of *T. urticae*.

## 1. Introduction

In recent decades, herbivorous spider mites (Acari: Tetranychidae), such as the two-spotted spider mite *Tetranychus urticae* Koch, which can attack a wide range of crops, have become some of the most important agricultural and horticultural pests worldwide [1,2]. In many crops, it is difficult or even impossible to control spider mites with acaricides because of their high mobility, high fecundity, high tolerance to chemicals, short life span, high costs of chemical control, ability to develop resistance to many classes of pesticides, and human safety issues [3]. Therefore, developing alternative control measures, such as biological control [4], is an important task.

Natural enemies of spider mites are employed to avoid the extensive increase of these pests in both indoor and outdoor environments. Numerous studies of acarophagous ladybirds [5,6], predatory anthocorids [7,8], predatory mites [9,10,11], and predatory thrips [12] have been conducted, envisioning the control of spider mites.

The ladybird *Stethorus gilvifrons* (Mulsant) (Coleoptera, Coccinellidae) is a highly voracious predator of spider mites in numerous agricultural and horticultural crops both in the open field and in greenhouses [13,14]. This species is widely distributed in Europe, Africa, and Asia [15]. Farahbakhsh [16] first reported this predator from Iran. Its general biological characteristics have been described [17,18] but only limited information is available about the effects of temperature on the life history of *S. gilvifrons* [19,20,21,22,23].

Abiotic conditions such as temperature are critical determinants of most biological processes, behavior, and ecological interactions of poikilothermous animals, such as insects and mites [24,25,26,27]. Temperature is an important ecological factor that affects the functions and efficacies of natural enemies of spider mites and can be used to predict changes in the population dynamics and phenology of insects and arachnids [28]. There exist only very few temperature-dependent development models for any *Stethorus* spp. [21,29].

Lower and upper threshold temperatures may influence pestiferous herbivores and their natural enemies in various ways, affecting growth, development, survival, and reproduction. Temperature-dependent development models permit the examination of the impacts of temperature on the local occurrence, geographic distribution, population dynamics, dormancy, overwintering, and management of insects and mites [30,31,32]. Knowledge of the performance of predators and parasitoids in natural and applied biological control at various temperature regimes is highly relevant in order to develop forecasting models and find the natural enemies that best-match with the environmental conditions of the target pests [33,34].

Numerous linear and non-linear models have been introduced to simulate the relationship between insect and arachnid development and temperature (Table 1). Linear models can estimate the lower-temperature threshold (*T*_0_) and the thermal constant (*K*) in a limited thermal range [29]. The developmental rate–temperature relationship in insects and mites is typically curvilinear (i.e., a dome-shape pattern) [35,36]. Non-linear models can describe the developmental rate over a wider range of temperatures and provide estimates of the lower and upper developmental thresholds (*T*_0_ and *T_max_*) and the optimum temperature (*T_opt_*) for insect or arachnid development. The main weakness of non-linear approaches is that the estimation of a thermal constant cannot be directly achieved, and some models cannot estimate *T*_0_ [37,38].

Using an existing dataset of temperature-dependent development of *S. gilvifrons* [23], here, our aim was to determine the thermal developmental trajectories and thermal thresholds of all immature stages of *S. gilvifrons* under semi-natural conditions by using linear and non-linear models. Such models are important by allowing the development of sampling and monitoring methods with simple rules for the timely detection and prediction of the presence and activity of the predators. Moreover, such models can be useful for developing mass-rearing techniques and estimating and comparing the dynamics and population growth of pests and their natural enemies.

## 2. Materials and Methods

### 2.1. Mite and Coccinellid Stock Colonies

The laboratory stock colonies of *T. urticae* and *S. gilvifrons* were established two months before starting the experiments with ~200 adult individuals of each species collected from two sugarcane fields (48°26′17.25″ E, 31°15′10.88″ N and 48°30′35.89″ E, 31°04′10.74″ N) located in Ahwaz region, Khuzestan province, Southwestern Iran [23]. *Tetranychus urticae* was reared on young maize plants (*Zea mays* var. KSC 704), which were grown from seeds in plastic pots (24 cm diameter and 26 cm depth) filled with a mixture of sandy loam, loam, and compost in equal proportions. No pesticides were used during plant growing. All plants were grown in a greenhouse at 27 ± 2 °C, 50 ± 10% RH, and natural photoperiod (~13–15:11–9 h L:D). The predatory beetles were reared in mesh covered aluminum cages (70 × 70 × 120 cm) on maize seedlings infested with spider mites. The cages were kept in the laboratory at 27 ± 1 °C and 50 ± 5% RH and natural photoperiod (~13–15:11–9 h L:D). The colony was maintained by the addition of spider mite-infested maize seedlings at weekly intervals. Additional maize plants were grown to produce leaves used in the life history experiments [23].

### 2.2. Experimental Design

To establish a cohort, twenty pairs of coccinellid females and males were incubated at 27 °C on maize leaf discs (8 cm diameter), with moist cotton pads provided as free water source, which were placed in a transparent plastic container (19 × 14 × 4 cm). Newly laid eggs of *S. gilvifrons* were individually transferred onto maize leaf squares (4 × 4 cm) resting on slightly moistened cotton pads in plastic dishes (6 cm diameter), which were stored in growth chambers set at 15, 20, 25, 27, 30, 34, and 38 °C, 50 ± 5% RH and a photoperiod of 16:8 h (L:D). The numbers of individuals (age < 24 h) at the beginning of the experiment were 200, 140, 120, 120, 120, 120, and 120 eggs at the above temperatures. Survivorship and the life stage of each individual at each temperature were checked and recorded daily. The different larval instars of *S. gilvifrons* were fed daily with *T. urticae* (about 100 mixed stages) and their life stages were determined based on their body size and the presence of the exuvium, which was used as the criterion of successful molting. Based on the recorded data, the incubation period, and the larval and pupal periods were determined for each individual. In addition, the total developmental time from the date of oviposition to adult emergence was calculated.

### 2.3. The Relationship between Temperature and Developmental Rate

Two linear and 20 non-linear models were examined to find the best-fitting model to describe the relationship between temperature and developmental rate of *S. gilvifrons* (Table 1). The common linear model or degree-day (DD) model, which is frequently used to determine the thermal constant (*K*) and lower temperature threshold, is as follows:

$$D_{r}=a+bT$$

where *T* is the temperature (°C), *D*_r_ is the development rate (days^−1^), *a* is the intercept, and *b* is the slope. The lower temperature threshold (*T*_0_) and the thermal constant (*K*, degree-days) were estimated using the parameters: *T*_0_ = −*a/b* and *K* = 1/*b* [35]. Ikemoto and Takai [39] proposed another linear model based on the development time formula as follows:

(DT)=K+tD

where *D* is developmental time (days), *T* is the temperature (°C), *t* is the lower temperature threshold, and *K* is the thermal constant. This equation is derived from the common linear model (1). The Ikemoto and Takai linear model represents a straight line with X = *T* and Y = *DT* [39].

The twenty nonlinear models (Table 1) were applied to estimate the lower and upper-temperature threshold and optimal temperature for development, each model was evaluated based on the following criteria:

1. The coefficient of determination (*R*^2^). A higher value *R*^2^ indicates better fit.
2. The residual sum of squares (*RSS*). A smaller value of *RSS* indicated better fit. The coefficient of determination and residual sum of the square is commonly used for model evaluation. However, the *R*^2^ value is not appropriate for discrimination between models with a different number of parameters because models with more parameters will provide a better fit. Therefore, we used the Akaike information criterion (*AIC*) and the adjusted coefficient of determination *R*^2^*_adj_*, which are independent of the number of parameters.

1. The Akaike information criterion. A good model must explicitly include smaller values of *AIC* [56,57], where *AIC* is

$$AIC=n\;ln\;\left[\frac{SSE}{n}\right]+2p$$

where *n* is the number of observations, *p* is the number of model parameters, including the intercept, and SSE is the sum of squared error.

2. The adjusted coefficient of determination (*R*^2^*_adj_*). A higher value of *R*^2^*_adj_* indicates better fit [38]. *R*^2^*_adj_* was calculated from the following equation:

$$R_{adj}^{2}=1-\left[\frac{n-1}{n-p}\right]\left(1-R^{2}\right)$$

where *n* is the number of observations, *p* is the number of model parameters, and *R*^2^ is the coefficient of determination or coefficient of nonlinear regression.

### 2.4. Statistical Analysis

#### 2.4.1. Linear Modeling

The raw life history data for the development of *S. gilvifrons* [23] were analyzed with the age-stage, two-sex life table [58,59] using the computer program TWO SEX-MSChart [60]. The variances and standard errors of the population parameters were estimated by the bootstrap technique [61], with 100.000 re-samplings to obtain stable estimates [62]. Then, paired bootstrap tests were used to examine the differences between the temperature treatments [23].

The relationship between temperature (*T*) and developmental rate (*r* = 1/d) for the immature stages was modeled using linear regression, where *r*(*T*) = a + b*T*, within the temperature range in which the relationship is linear (15 to 34 °C). The model was fitted using Microsoft Office Excel 2007. The lower developmental threshold temperature was estimated by extrapolating the regression line to the temperature axis, *t_b_* = −a/b. In addition, degree-day estimations for development were calculated using the formula *K* = (*T* − *t_b_*) Dev, where *K* is degree-days, Dev is the mean number of days to complete development at a constant temperature (*T*), and *t_b_* is the lower threshold temperature [63].

#### 2.4.2. Non-Linear Modeling

Temperature-dependent mean rates of development, *r*(*T*), were modeled using twenty descriptive nonlinear models (Table 1). The parameters of the non-linear models were estimated with the non-linear regression model of Marquardt [64] using the JMP (IN 4.lnk) and Excel (version 2007) programs. Lower and upper developmental thresholds were estimated from the equations.

## 3. Results

### 3.1. Developmental Time (Data from *[23]*)

The predators completed development from egg to adult at all temperatures examined, except at 38 °C (Table 2). At 38 °C, all eggs failed to hatch. The duration of the immature stages decreased sharply with increasing temperature. The mean incubation period decreased with increasing temperature up to 34 °C. The developmental period of eggs ranged from 15.7 and 15.7 d at 15 °C to 3.7 and 3.5 d at 34 °C for females and males, respectively. Similarly, the larval period was affected significantly by temperature and the shortest larval period was observed at 34 °C. There were no statistically significant differences between the larval periods at 25 and 27 °C for females and at 25, 27, and 30 °C for males. Pupal development ranged from a mean of 11.8 and 12.1 days at 15 °C to 2.6 and 2.9 days at 34 °C for females and males, respectively.

### 3.2. Model Evaluation

#### 3.2.1. Linear Models

**Figure 1:**
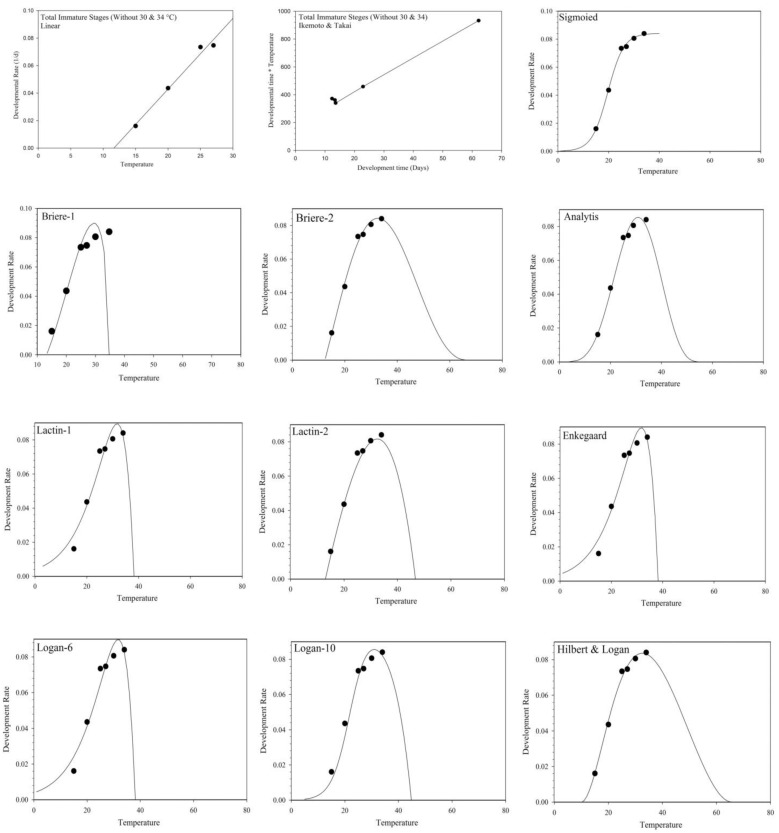

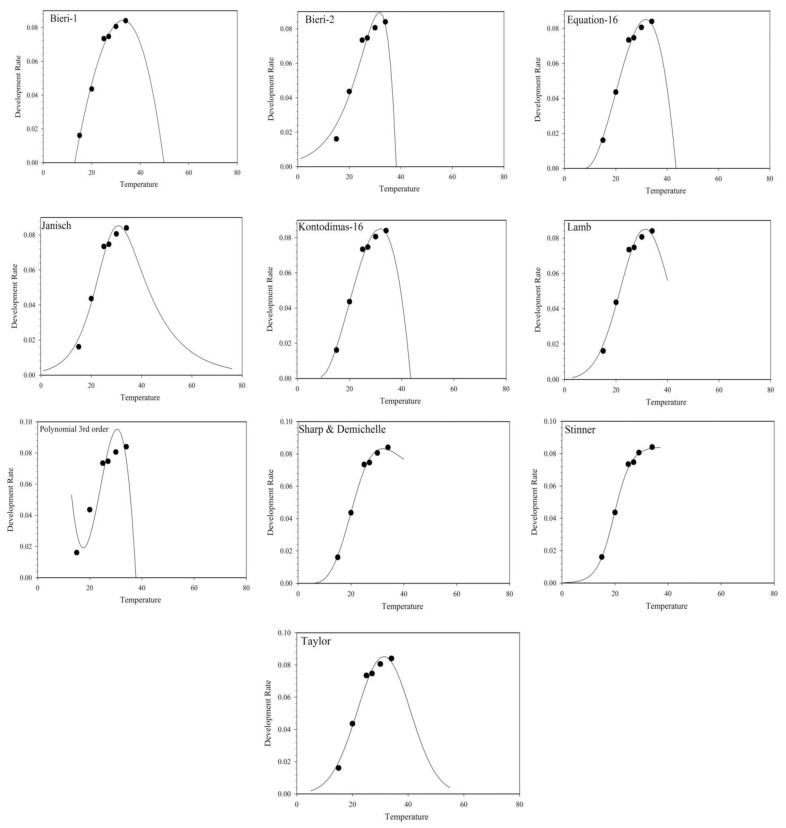
Fitting two linear and twenty non-linear models to the observed developmental rates (1/d) of all immature stages at six constant temperatures (°C). Dots represent the observed data [23].

The common linear model and the Ikemoto and Takai model were applied only to data within the temperature range of 15 to 27 °C since the data at 30 and 34 °C deviated from linearity. Both linear models fitted the stage-specific developmental rate well (Figure 1) and showed an acceptable fit to data for all immature stages combined. The linear regression equation, the lower-temperature threshold (*T_min_*), and the thermal constant (*K*) of *S. gilvifrons* were calculated for each immature stage (Table 3). Neither of the two linear models provides an optimal temperature or an upper-temperature threshold. The Ikemoto and Takai linear model had higher *R*^2^ and *R*^2^*_adj_* values than the common linear model (Table 3), indicating a slightly higher degree of confidence in parameter estimates provided by the Ikemoto and Takai model. The Ikemoto and Takai model tended to give higher estimates of *T_min_* and lower estimates of the thermal constant than the common linear model for egg to pupa development (Table 3).

#### 3.2.2. Non-Linear Models

All twenty models (Sigmoid, Logan-6, Logan-10, Lactin-1, Lactin-2, Briere-1, Briere-2, Polynomial 3rd order, Kontodimas-16, Janisch, Taylor, Stinner, Hilbert & Logan (Holling III), Lamb, Analytis, Equation-16, Enkegaard, Bieri-1, Bieri-2, and Sharp & DeMichele) fitted the immature developmental rate well at the temperature range from 15 to 34 °C (Table 4; Table 5; Table 6; Figure 1).

The values of *R*^2^, *RSS*, *AIC*, and *R*^2^*_adj_* used to evaluate the goodness-of-fit for each model showed that Briere-2, with the highest values for *R*^2^ and *R*^2^*_adj_* and the lowest values for *RSS* and *AIC,* had the best fit to the development of each immature stage and total immature development, and was thus used to estimate the lower-temperature threshold and the optimal temperature. Logan-10 was used to estimate the upper-temperature threshold. The Briere-1 model had the poorest fit to data among all twenty non-linear models (Table 5).

## 4. Discussion

This study is the first to model temperature-dependent development and the thermal requirements of the ladybird *S. gilvifrons.* By providing fundamental information on the thermal biology of this predator, this study may benefit implementation of the use of this predator as biological control agent of spider mites.

The study underlying the modeling presented here [23] demonstrated that the temperature-specific developmental times of *S. gilvifrons* are sex-specific. There were small but significant differences between the development times from egg to adult measured for females (61.4, 31.6, 14.8, 13.3, 12.5, and 11.7 days) and those for males (63.2, 28.5, 13.2, 13.4, 12.2, and 12.3 days) at 15, 20, 25, 27, 30, and 34 °C [23]. Interestingly, males developed more slowly than females at the extremes, at 15 and 34 °C, but somewhat faster at moderate temperatures 20, 25, and 30 °C. These results slightly differ from the total developmental time estimated by Taghizadeh [20] for *S. gilvifrons* (56.4, 31.1, 18.5, 17.5, 12.4, and 9.2 days at 15, 20, 2, 2, 3, and 35 °C). Roy [29] reported that *S. punctillum* successfully developed between 14 and 34 °C, with 68.5 and 12.1 days, respectively, but egg hatching failed at 12 and 36 °C. Jafari [23] showed that the total developmental time of *S. gilvifrons* on *T. urticae* at 25 °C was 14.4 for females and 13.2 days for males, whereas Taghizadeh [20] and Aksit [65] observed 18.5 days and 14.6 days at the same temperature. Roy [29] determined this parameter for *S. punctillum* on *T. mcdanieli* at 24 °C to be 17.1 days, Shih [66]) for *S. loi* on *T. kanzawai* at 23.8 °C to be 15.2 days, Fiaboe [67] for *S. tridense* on *T. evansi* at 24 °C to be 17.4 days, Khan and Spooner-Hart [68] for *S. vagans* on *T. urticae* at 25 °C to be 13.1 days, and Mori [69] for *S. japonicus* on *T. urticae* at 25 °C to be 17.1 days. Apart from species-specific characteristics, differences among these studies might also be due to different rearing techniques and experimental conditions, host plants used in the experiments, prey mite species, photoperiod, along with differences in data analysis. Taken together, the total developmental duration in the range of 25 to 27 °C was 19 to 13 days for both females and males in all the different *Stethorus* spp. tested [70,71,72,73].

The common linear model [35] has been widely used for its simplicity and suitability to calculate the thermal constant (*K*) [37,74]. When comparing the two linear models using the *R*^2^ and *R*^2^*_adj_* coefficients, the Ikemoto and Takai model indicated a better fit for the development of all immature stages of *S. gilvifrons* than the common linear model and was thus used to estimate *T*_0_ and the thermal constant. In line with previous studies, demonstrating a similar lower-temperature threshold in *Stethorus* spp., no development was observed at 10–12 °C [21,29,67,69], with development starting at around 15 °C among the temperatures tested. Estimation of the lower-temperature thresholds in our study revealed around 12 °C *T*_0,_ using the common linear and Ikemoto and Takai models (Table 3). It remains to be shown whether fluctuating temperatures exert different effects to constant temperatures [75] on the development and lower thermal threshold of *S. gilvifrons*. Pertinent studies on encyrtid and eulophid parasitoids found indeed differences in developmental times and lower thresholds between fluctuating and constant temperature regimes [76,77]. Moreover, the vapor pressure deficit, which changes with temperature at the same ambient relative humidity, might be a factor in temperature-specific developmental rates (e.g., [78]).

The estimated *T*_0_ and thermal constant (11.96 °C and 187.87 DD, respectively) estimated by the Ikemoto linear model for total development (Table 3) were lower than the corresponding values reported for *S. gilvifrons* (12.47 °C and 222.72 DD) estimated by the common linear model [21].

Most non-linear models used in our study showed a statistically highly significant goodness of fit according to *R*^2^, *R*^2^*_adj_*_,_ *RSS*, and *AIC* values. Therefore, in addition to the above, the temperature-related biological parameters *T*_0_, *T_opt_* and *T_max_* were used to select the best model for describing the relationship between the developmental rates of *S. gilvifrons* and temperature. The Sigmoid, Logan-6, Logan-10, Lactin-1, Janisch, Stinner, Lamb, Polynomial 3rd-order, Sharpe and DeMichele, Taylor, and Bieri-2 models could not be used to estimate *T*_0_ because there was no intersection with the temperature axis ( Table 4; Figure 1).

The Polynomial 3rd-order, Equation-16, Kontodimas-16, and Analytis models overestimated *T_max_* for *S. gilvifrons* in all immature stages and some provided an inaccurate estimation of the lower-temperature threshold. In contrast, the Sigmoid, Lamb, Janisch, Stinner, Sharpe and DeMichele, and Taylor models underestimated *T_max_*. The estimated *T_opt_* by the Janisch model was lower than the experimental values for all immature stages. Among all non-linear models, the Briere-2 model was the most efficient for the description of temperature-dependent development of the predators regarding *T*_0_ and *T_opt_*. The Logan-10 model provided the best estimate of *T_max_* (Table 6). Roy [29] determined an optimum temperature of about 30 to 32 °C using the Briere-1 model for *S. punctillum*. In our study, the optimal temperature for the development of *S. gilvifrons* was about 33 °C for total development and the upper-thermal threshold varied between 38 and 44 °C depending on the model, which indicates that these predators can thrive under the same broad range of temperatures (15–37 °C) as their prey *T. urticae*. Several studies have shown that the optimal temperature for *T. urticae* is around 35 °C and that the lower- and upper-temperature thresholds are about 10 and 38 °C, respectively [79,80,81,82,83]. Afshari [84] reported that *S. gilvifrons* successfully developed between 35.25 to 37.80 °C during the warm growing season (May–September), in the sugarcane fields of Ahvaz, Southwestern Iran. While these field observations suggested that *S. gilvifrons* well tolerates higher temperatures (up to 37 °C), our study shows that the upper-temperature threshold estimated by the Logan-10 model lies between 36.97 and 44.03 °C (Table 6), or at about 40 °C, calculated by other non-linear models [21]. In biological control, the estimation of the temperature thresholds, thermal constants, and optimal temperature for the development of natural enemies can substantially contribute to the selection of the most suitable natural enemy to be used under different environmental conditions [6,29,85].

The results of our study indicate that *S. gilvifrons* can be a promising biological control agent for spider mites over a broad range of temperatures that are common in subtropical and tropical climates. *Stethorus* spp. are distributed in many different climates, ranging from tropical (*S. gilvifrons*, *S. tridens* and *S. loi*) [20,21,23,65,66] to temperate (*S. punctillum*, *S. japonicus*, *S. vagans* and *S. pauperculus*) [29,68,69,70]. All *Stethorus* spp. occurring in temperate and subtropical climates overwinter in the adult stage, whereas tropical species seldomly enter diapause [15]. Degree-day models developed from constant temperature experiments can be used to predict the emergence of *S. gilvifrons* in the field. Combining estimates from linear and non-linear models provides for a useful prediction of the first emergence of adult *S. gilvifrons* during the vegetative season. Undoubtedly, the development rate of *S. gilvifrons* is also influenced by factors other than temperature, such as nutrition, humidity, and photoperiod, which were not included in the models presented here. The parameter estimates obtained by our models were derived from laboratory studies conducted at strictly defined climatic conditions [23], while in natural and agricultural environments the predators are subjected to more complex and fluctuating conditions. Nevertheless, the models presented here indicate that *S. gilvifrons* is also a promising candidate for utilization as a biological control agent at more extreme low and high temperatures, as compared to other predators of *T. urticae,* in terms of temperature tolerance [19,23,86,87,88,89]. Estimating temperature-dependent development and modeling the thermal requirements of *S. gilvifrons* is important for creating forecasting models. Understanding its thermal adaptations allows to predict the temporal synchrony with its prey and to choose the best time for releasing the predators under both field and greenhouse conditions.

## Figures and Tables

**Table 1:** Linear and non-linear models used to simulate the developmental rates of *S. gilvifrons* at various constant temperatures.

Equation	Model	References
D(T)=a+bT $T_{0}=-\frac{a}{b}$ $\;\;\;\;K=\frac{1}{b}$	Ordinary linear regression	[35]
$DT=K+t_{min}D$	Ikemoto & Takai	[39]
$R\left(T\right)=\frac{c}{1+e^{\left(a+b.T\right)}}$	Sigmoid	[40]
$R\left(T\right)=\Psi \times \left[\begin{array}{c} e^{\rho \times Τ}-e^{\left(\rho \times T_{max}-\frac{T_{max}-T}{\Delta T}\right)} \end{array}\right]$	Logan-6	[41]
$R\left(T\right)=a\times \left[\begin{array}{c} \frac{1}{1+K\times e^{-\rho \times T}}-\;e^{\left(\frac{T_{max}-T}{\Delta T}\right)\;\;} \end{array}\right]$	Logan-10	[41]
$R\left(T\right)=e^{\rho \times Τ}-e^{\left(\rho \times T_{max}-\left(\frac{T_{max}-T}{\Delta T}\right)\right)}$	Lactin-1	[42]
$R\left(T\right)=e^{\rho \times Τ}-e^{\left(\rho \times T_{max}-\left(\frac{T_{max}-T}{\Delta T}\right)\right)+\lambda }$	Lactin-2	[42]
$R\left(T\right)=a\times T\left(T-T_{0}\right)\times \sqrt{T_{max}-T}$	Briere-1	[43]
$R\left(T\right)=a\times T\left(T-T_{0}\right)\times \sqrt[{m}]{T_{max}-T}$	Briere-2	[43]
$R\left(T\right)=a{\left(T-T_{a}\right)}^{n\;\times \;}\;{\left(T_{\max}-T\right)}^{m}$	Analytis	[44,45]
$R\left(T\right)=ax^{3}+bx^{2}+cx+d$	Polynomial 3rd order	[46]
$R\left(T\right)=a{\left(T-T_{a}\right)}^{2}\left(T_{max}-T\right)$	Kontodimas-16	[37]
$D\left(T\right)=\frac{2}{D_{min}\left(e^{k\left(T-T_{opt}\right)}+e^{-\lambda \left(T-T_{opt}\right)}\right)}$	Janisch	[47]
$D\left(T\right)=R_{m}^{e\left(-as\times {\left(\frac{T-T_{m}}{T_{a}}\right)}^{a}\right)}$	Taylor	[48]
$R\left(T\right)=\frac{c}{1+e^{a+b\times T}}\;\;\;for\;\;T\;\leq T_{opt}$ $R\left(T\right)=\frac{c}{1+e^{a_{1}+b_{1}\left(2\times T_{opt}-T\right)}}\;for\;T>T_{opt}$	Stinner	[49]
$R\left(T\right)=\Psi \left[\frac{\left(T-T_{0}\right){\;}^{2}}{{\left(T-T_{0}\right)}^{2\;\;}+D^{2}}-e^{\left(-\frac{T_{\max}-\left(T-T_{0}\right)}{\Delta }\right)}\right]$	Hilbert & Logan, or Holling III	[50,51]
$R\left(T\right)=R_{m}\times \exp\left[-0.05{\left(\frac{T-T_{opt}}{T_{0}}\right)}^{2}\right]\;\;\;for\;T\leq \;T_{opt}$ $R\left(T\right)=R_{m}\times \exp\left[-0.05{\left(\frac{T-T_{opt}}{T_{max}}\right)}^{2}\right]\;\;for\;T>T_{opt}$	Lamb	[52]
$\frac{1}{D}=a{\left(T-t_{min}\right)}^{2}\;\left(t_{max\;}-T\right)$	Equation-16	[37]
$\frac{1}{D}=\left(a+b\times T\right)\times \left[e\left(-\left(c+d\times T\right)\right)\right]$	Enkegaard	[53]
$\frac{1}{D}=\left[a\left(T-x_{max}\right)\right]-\left[b^{\left(T-x_{min}\right)}\right]$	Bieri-1	[54]
$\frac{1}{D}=a\left(\frac{\left(T-x_{min}\right)}{b^{\left(T-x_{min}\right)}}\right)$	Bieri-2	[54]
$R\left(T\right)=\frac{\varphi \frac{T}{T_{1}}\;exp\left[\frac{\Delta H_{A}}{R}\;\;\left(\frac{1}{T_{1}}-\frac{1}{T}\right)\right]\;\;\;\;\;\;\;\;}{1+exp\left[\frac{\Delta H_{L}}{R}\;\;\left(\frac{1}{T_{L}}-\frac{1}{T}\right)\right]+exp\left[\frac{\Delta H_{H}}{R}\left(\frac{1}{T_{H}}-\frac{1}{T}\right)\right]}$	Sharpe & DeMichele	[55]

**Table 2:** Female and male immature developmental time (mean ± SE, days; *n* is the number of replicates) of *Stethorus gilvifrons* at six constant temperatures (data from [23]).

Sex	Stage				Temperatures (°C)								
		15	*n*	20	*n*	25	*n*	27	*n*	30	*n*	34	*n*
Female	Egg	15.7 ± 0.5 ^a^	139	8.7 ± 0.2 ^b^	125	3.3 ± 0.0 ^d^	115	3.2 ± 0.1 ^de^	118	3.0 ± 0.0 ^e^	118	3.7 ± 0.0 ^c^	92
	Larva	33.9 ± 0.7 ^a^	17	15.1 ± 0.4 ^b^	49	7.5 ± 0.3 ^c^	70	7.2 ± 0.1 ^c^	79	6.4 ± 0.2 ^d^	70	5.3 ± 0.1 ^e^	59
	Pupa	11.8 ± 0.2 ^a^	17	7.7 ± 0.4 ^b^	39	3.5 ± 0.1 ^c^	64	2.8 ± 0.0 ^d^	62	2.9 ± 0.1 ^e^	62	2.6 ± 0.0 ^de^	58
Male	Egg	15.7 ± 0.5 ^a^	139	7.3 ± 0.1 ^b^	125	3.8 ± 0.1 ^c^	115	3.5 ± 0.1 ^c^	118	3.0 ± 0.0 ^d^	118	3.5 ± 0.1 ^c^	92
	Larva	35.4 ± 1.0 ^a^	17	14.4 ± 0.3 ^b^	49	6.6 ± 0.3 ^cd^	70	7.0 ± 0.2 ^c^	79	6.3 ± 0.1 ^d^	70	5.8 ± 0.1 ^e^	59
	Pupa	12.1 ± 0.2 ^a^	17	6.6 ± 0.3 ^b^	39	2.8 ± 0.1 ^c^	64	2.9 ± 0.0 ^c^	62	2.9 ± 0.0 ^c^	62	2.9 ± 0.0 ^c^	58

Means followed by different letters within each row are significantly different according to the paired bootstrap test at a 95% confidence interval. The SEs were estimated by 100.000 bootstraps.

**Table 3:** Linear regression equations, lower temperature threshold (*T_min_*), and thermal constant (degree-days) of *S. gilvifrons* immature stages using two linear models.

Linear Model	Stage	Thermal Range	Linear Equation	Thermal Constant (DD) ± SE (°C)	*T_min_* ± SE (°C)	*R* ^2^	*R* ^2^ *_adj_*	*p*
Common	Egg	15–27	DR = 0.0208T − 0.261	48.02 ± 12.76	12.55 ± 0.73	0.9610	0.9415	0.019
	Larva	15–27	DR = 0.0103T − 0.129	96.95 ± 14.51	12.51 ± 0.27	0.9509	0.9263	0.025
	Pupa	15–27	DR = 0.0240T − 0.295	41.70 ± 22.10	12.33 ± 0.70	0.9608	0.9413	0.019
	Egg-Pupa	15–27	DR = 0.0051T − 0.059	194.50 ± 36.04	11.64 ± 0.29	0.9816	0.9724	0.009
Ikemoto & Takai	Egg	15–27	DT = 11.79D + 53.25	53.25 ± 0.008	11.79 ± 0.46	0.9807	0.9711	0.009
	Larva	15–27	DT = 12.16D + 102.34	102.34 ± 0.004	12.16 ± 0.61	0.9941	0.9911	0.002
	Pupa	15–27	DT = 11.58D + 46.01	46.01 ± 0.009	11.58 ± 0.40	0.9777	0.9661	0.011
	Egg-Pupa	15–27	DT = 11.96D + 187.87	187.87 ± 0.002	11.96 ± 0.51	0.9951	0.9934	0.0006

**Table 4:** Parameter estimates for the twenty non-linear, temperature-dependent models used for describing immature development of *S. gilvifrons*.

Model	Parameters	Egg	Larva	Pupa	Total Development
Briere-1	*a*	30.51 × 10^−5^	17.50 × 10^−5^	35.61 × 10^−5^	7.86 × 10^−5^
	*t_min_*	13.48	14.38	13.64	13.00
	*t_opt_*	29.75	29.75	29.75	29.75
	*t_max_*	35.00	35.00	35.00	35.00
Briere-2	*a*	3.55 × 10^−4^	1.67 × 10^−6^	1.82 × 10^−4^	6.30 × 10^−9^
	*m*	3.09	0.64	1.68	0.358
	*t_min_*	11.17	12.20	11.24	11.96
	*t_opt_*	31.00	35.75	31.50	32.00
	*t_max_*	34.93	57.80	38.80	67.48
Logan-6	φ	−0.047	1.066	−0.045	0.570
	ρ	0.185	0.166	0.167	0.151
	Δ	6.83	5.59	6.09	6.41
	*t_opt_*	30.75	32.50	31.75	31.75
	*t_max_*	36.22	38.68	37.55	38.21
Logan-10	α	0.466	0.517	0.419	0.105
	ρ	0.236	0.176	0.315	0.291
	Δ	3.71	7.60	4.67	6.49
	*k*	249.56	138.09	847.63	586.62
	*T_L_*	38.66	44.05	44.05	44.04
	*t_opt_*	33.75	34.25	31.00	32.75
	*t_max_*	38.43	44.03	36.97	44.03
Lactin-1	Δ	5.4453	6.1696	6.0314	6.5082
	ρ	0.1834	0.1619	0.1654	0.1535
	*t_opt_*	31.00	33.00	31.09	32.00
	*t_max_*	36.3378	38.7646	37.5502	38.2170
Lactin-2	Δ	2.70	9.87	5.01	26.62
	ρ	0.01	0.011	0.018	0.017
	*e*	1.21	1.13	1.24	0.86
	*T_L_*	39.87	59.56	44.67	70.78
	*t_min_*	12.50	12.48	12.09	13.04
	*t_opt_*	30.75	34.00	31.50	32.25
	*t_max_*	36.50	47.27	36.62	46.73
Sigmoid	*a*	7.6721	5.8402	6.5996	6.2261
	*b*	−0.3775	−0.2714	−0.3224	−0.3167
	*c*	0.3105	0.1824	0.3772	0.0842
Hilbert & Logan, or Holling III	φ	0.8069	1.1954	2.6544	1.4284
	*T*_0_	5.1643	−0.0286	2.2864	−5.4646
	*d*	2.6455	34.8905	10.2500	62.5963
	*T_L_*	33.6489	71.6097	46.7278	75.7471
	Δ	17.1084	20.7993	20.0060	19.6420
	*t_min_*	6.18	7.3623	11.2824	12.4085
	*t_opt_*	31.00	36.11	31.18	33.00
	*t_max_*	38.21	67.99	45.91	66.11
Stinner (*T* > *T_opt_*)	*a*	−2.9618	−1.7579	−2.4292	−2.6428
	*b*	0.1898	0.1355	0.1612	0.1583
	*c*	0.3102	0.1824	0.3772	0.0842
	*t_opt_*	-	-	-	-
Polynomial 3rd order	*a*	1.5108	0.0316	0.9548	0.8113
	*b*	−0.2282	−0.0137	−0.1512	−0.1118
	*c*	0.0112	0.0011	0.0079	5.02 × 10^−3^
	*d*	−1.65 × 10^−4^	−1.90 × 10^−5^	−1.18 × 10^−4^	−6.97 × 10^−5^
	*t_opt_*	30.00	34.25	31.42	32.50
	*t_max_*	38.00	47.47	40.57	37.69
Equation-16	*a*	8.9669 × 10^−5^	2.2398 × 10^−5^	6.9155 × 10^−5^	1.2974 × 10^−5^
	*t_min_*	10.7196	8.7399	9.2019	8.0642
	*t_opt_*	30.00	34.00	31.40	32.00
	*t_max_*	39.6390	46.5227	42.4350	43.4616
Kontodimas-16	*a*	8.9669 × 10^−5^	2.2398 × 10^−5^	6.9155 × 10^−5^	1.2973 × 10^−5^
	*t_min_*	10.7196	8.7399	9.2019	8.0643
	*t_opt_*	30.00	34.01	31.19	31.90
	*t_max_*	39.6390	46.5227	42.3350	43.4615
Lamb (*T* > *Topt)*	*R_m_*	0.3245	0.1780	0.3782	0.0852
	*t_m_* (=*t_opt_*)	29.6197	33.1083	30.8630	31.2873
	*T*_0_	7.4244	9.7908	8.6507	9.5089
Analytis	*a*	1.3038 × 10^−15^	1.1464 × 10^−15^	1.3474 × 10^−14^	2.2536 × 10^−17^
	*n*	7.6175	6.1802	5.3244	5.8251
	*m*	2.1428	2.5713	3.9925	4.7378
	*t_min_*	−9.4633	−17.2948	−0.3747	−1.9163
	*t_opt_*	30.10	37.40	31.02	31.00
	*t_max_*	41.1822	60.0903	54.5616	57.4548
Enkegaard	*a*	0.0248	0.0178	0.0407	0.0122
	*b*	−68.3956 × 10^−5^	−46.1022 × 10^−5^	−10.8593 × 10^−4^	−31.9438 × 10^−5^
	*c*	1.1245	1.1075	1.1075	1.1075
	*d*	−0.1835	−0.1620	−0.1656	−0.1535
	*t_opt_*	31.11	33.00	31.90	32.01
	*t_max_*	36.66	38.76	37.55	38.21
Taylor	*R*_*m*_	0.3245	0.1780	0.3782	0.0852
	*t_m_* (=*t_opt_*)	29.6197	33.1083	30.8630	31.2874
	$T_{\sigma }$	7.4244	9.7909	8.6509	9.5093
Janisch	*D*_*min*_	3.0985	5.6899	3.6422	12.3639
	*k*	−0.1834	−0.1414	−0.2354	−0.1547
	λ	0.1221	−0.0975	−0.0364	−0.0785
	*t_opt_*	28.0868	30.7786	23.6969	27.90
Sharpe & DeMichele	*a*	5.1991	−10.5143	−5.2836	−8.4871
	*b*	−322.3149	−1502.5915	−259.0272	−1360.8376
	*c*	7.9817	−6.6145	−2.8555	−4.5433
	*d*	−365.7124	−1536.7211	−304.9700	−1403.3453
	*f*	17.7782	0.1576	3.076	0.1380
	*g*	−38.2689	−1276.7436	−108.8371	−1252.6541
	*t_opt_*	30.00	32.23	31.00	32.05
Bieri-1	*a*	0.0209	0.103	0.0249	0.0177
	*b*	1.5436	1.3103	1.274	1.0288
	*x*_*max*_	12.5366	12.4235	12.2484	−6.9928
	*x*_*min*_	37.99	45.5230	40.99	49.43
	*t_min_*	12.69	12.52	12.32	12.96
	*t_opt_*	31.01	33.50	32.00	33.04
	*t_max_*	36.33	41.03	39.38	49.76
Bieri-2	*a*	−0.1749	−0.0813	−0.1801	−0.0373
	*b*	0.8323	0.8504	0.8473	0.8575
	*x*_*min*_	36.33	38.76	37.55	38.21
	*t_opt_*	31.00	33.00	31.53	32.00
	*t*_*max*_	36.33	38.76	37.55	38.21

**Table 5:** Goodness-of-fit measures for the twenty non-linear, temperature-dependent models used for describing immature development of *S. gilvifrons*.

Model	Parameters	Egg	Larva	Pupa	Total Development
Briere-1	*R* ^2^	0.9177	0.6358	0.8053	0.7871
	*R^2^_adj_*	0.8628	0.3930	0.6755	0.6451
	*AIC*	−35.69	−35.49	−28.75	−46.31
	*RSS* (10^−4^)	57.63	83.13	183.13	72.60
Briere-2	*R* ^2^	0.9773	0.9716	0.9627	0.9923
	*R^2^_adj_*	0.9432	0.9290	0.9069	0.9807
	*AIC*	−42.72	−47.75	−38.04	−65.33
	*RSS* (10^−4^)	12.79	5.53	27.90	0.29
Logan-6	*R* ^2^	0.9634	0.9444	0.9447	0.9493
	*R^2^_adj_*	0.9085	0.8661	0.8617	0.8732
	*AIC*	−39.55	−44.16	−35.46	−54.10
	*RSS* (10^−4^)	21.70	10.05	42.90	1.92
Logan-10	*R* ^2^	0.9903	0.9757	0.9785	0.9956
	*R^2^_adj_*	0.9515	0.8785	0.8925	0.9780
	*AIC*	−47.67	−49.33	−41.12	−69.08
	*RSS* (10^−4^)	5.60	4.25	16.69	1.34
Lactin-1	*R* ^2^	0.9735	0.9696	0.9586	0.9919
	*R^2^_adj_*	0.9559	0.9494	0.9311	0.9154
	*AIC*	−43.52	−46.19	−37.45	−56.10
	*RSS* (10^−4^)	15.61	9.99	42.90	1.91
Lactin-2	*R* ^2^	0.9576	0.9648	0.9587	0.9776
	*R^2^_adj_*	0.8940	0.9120	0.8967	0.9440
	*AIC*	−31.02	−47.04	−37.41	−57.99
	*RSS* (10^−4^)	89.97	6.22	30.98	0.61
Sigmoid	*R* ^2^	0.9362	0.9777	0.9731	0.9969
	*R^2^_adj_*	0.8936	0.9629	0.9552	0.9948
	*AIC*	−38.03	−51.85	−41.50	−73.20
	*RSS* (10^−4^)	38.96	3.89	21.85	0.11
Hilbert & Logan, or Holling III	*R* ^2^	0.9792	0.9737	0.9697	0.9937
	*R^2^_adj_*	0.8963	0.8685	0.8485	0.9686
	*AIC*	−41.32	−46.84	−37.22	−64.80
	*RSS* (10^−4^)	11.57	4.61	22.91	0.23
Stinner (*T* > *Topt*)	*R* ^2^	0.9362	0.9777	0.9731	0.9969
	*R^2^_adj_*	0.8406	0.9440	0.9329	0.9949
	*AIC*	−36.03	−49.85	−39.50	−71.20
	*RSS* (10^−4^)	38.96	3.89	21.85	0.11
Polynomial 3rd order	*R* ^2^	0.9840	0.9714	0.9728	0.9924
	*R^2^_adj_*	0.9600	0.9285	0.9321	0.9810
	*AIC*	−43.44	−48.35	−39.94	−65.87
	*RSS* (10^−4^)	11.34	5.01	20.34	0.27
Equation-16	*R* ^2^	0.9696	0.9712	0.9661	0.9880
	*R^2^_adj_*	0.9493	0.9521	0.9436	0.9801
	*AIC*	−43.24	−50.31	−40.54	−65.08
	*RSS* (10^−4^)	0.16	5.03	25.63	0.42
Kontodimas-16	*R* ^2^	0.9723	0.9712	0.9659	0.9880
	*R^2^_adj_*	0.9539	0.9521	0.9433	0.9801
	*AIC*	−43.24	−50.13	−40.54	−65.08
	*RSS* (10^−4^)	16.35	5.03	25.63	0.42
Lamb (*T* > *Topt)*	*R* ^2^	0.9870	0.9674	0.9735	0.9825
	*R^2^_adj_*	0.9783	0.9456	0.9559	0.9708
	*AIC*	−35.68	−49.51	−42.08	−62.64
	*RSS* (10^−4^)	57.64	5.76	19.85	0.64
Analytis	*R* ^2^	0.9878	0.9517	0.9718	0.9848
	*R^2^_adj_*	0.9493	0.7587	0.8592	0.9241
	*AIC*	−44.37	−42.41	−37.68	−58.50
	*RSS* (10^−4^)	6.95	9.62	21.23	0.55
Enkegaard	*R* ^2^	0.9735	0.9450	0.9447	0.9492
	*R^2^_adj_*	0.9339	0.8625	0.8618	0.8732
	*AIC*	−41.52	−44.19	−35.45	−54.10
	*RSS* (10^−4^)	15.61	9.90	42.90	1.91
Taylor	*R* ^2^	0.9870	0.9674	0.9735	0.9825
	*R^2^_adj_*	0.9783	0.9456	0.9559	0.9708
	*AIC*	−47.52	−49.51	−42.08	−62.64
	*RSS* (10^−4^)	8.02	5.76	19.85	0.64
Janisch	*R* ^2^	0.9928	0.9595	0.9821	0.9743
	*R^2^_adj_*	0.9821	0.8988	0.9553	0.9358
	*AIC*	−49.55	−45.92	−42.25	−58.34
	*RSS* (10^−4^)	4.09	7.49	13.83	0.95
Sharpe & DeMichele	*R* ^2^	0.9875	0.9668	0.9759	0.9939
	*R^2^_adj_*	*	*	*	*
	*AIC*	−42.09	−45.54	−40.50	−63.08
	*RSS* (10^−4^)	7.29	5.82	18.51	0.22
Bieri-1	*R* ^2^	0.9700	0.9676	0.9565	0.9915
	*R^2^_adj_*	0.9250	0.9190	0.8913	0.9792
	*AIC*	−41.04	−47.59	−37.11	−65.24
	*RSS* (10^−4^)	16.90	5.67	32.65	0.29
Bieri-2	*R* ^2^	0.9735	0.9449	0.9447	0.9492
	*R^2^_adj_*	0.9559	0.9083	0.9079	0.9154
	*AIC*	−43.52	−49.59	−39.11	−56.10
	*RSS* (10^−4^)	15.61	9.99	42.90	1.91

* Non-calculable because the number of observations equals the number of model parameters, which results in a denominator of 0 in the equation.

**Table 6:** Estimated optimal temperature, temperature thresholds, and models applied to estimate these parameters for different immature stages and total development of *S. gilvifrons*.

Life Stage	*T_min_* ± SE (°C)	Model	*T_opt_* ± SE ^a^ (°C)	Model	*T_max_* ± SE (°C)	Model
Egg	11.17 ± 3.0183	Briere-2	31.00	Briere-2	38.43 ± 3.40	Logan-10
Larva	12.20 ± 3.7379	Briere-2	35.75	Briere-2	44.03 ± 3.74	Logan-10
Pupa	11.24 ± 4.2108	Briere-2	31.50	Briere-2	36.97 ± 4.98	Logan-10
Total development	11.96 ± 1.3177	Briere-2	32.00	Briere-2	44.03 ± 3.52	Logan-10

^a^ SE could not be estimated because *T_opt_* in this model was calculated using the graphical method.

## Data Availability

The data presented in this study are available on request from the first author (M.J.).

## References

[1] Helle W., Sabelis M.W. (1985). Spider Mites: Their Biology, Natural Enemies, and Control.

[2] Khanamani M., Fathipour Y., Hajiqanbar H., Sedaratian A. (2014). Two-spotted spider mite reared on resistant eggplant affects consumption rate and life table parameters of its predator, *Typhlodromus bagdasarjani* (Acari: Phytoseiidae). Exp. Appl. Acarol..

[3] Attia S., Grissa-Lebdi K., Lognay G., Bitume E., Hance T., Mailleux A.C. (2013). A review of the major biological approaches to control the worldwide pest *Tetranychus urticae* (Acari: Tetranychidae) with special reference to natural pesticides. J. Pest Sci..

[4] Van Leeuwen T., Vontas J., Tsagkarakou A., Dermauw W., Tirry L. (2010). Acaricide resistance mechanisms in the two-spotted spider mite *Tetranychus urticae* and other important Acari: A review. Insect Biochem. Mol. Biol..

[5] Obrycki J.J., Kring T.J. (1998). Predaceous coccinellidae in biological control. Annu. Rev. Èntomol..

[6] Roy M., Brodeur J., Cloutier C. (2003). Effect of temperature on intrinsic rates of natural increase (rm) of a coccinellid and its spider mite prey. Biocontrol.

[7] Madadi H., Enkegaard A., Brødsgaard H.F., Kharrazi-Pakdel A., Ashouri A., Mohaghegh-Neishabouri J. (2009). Interactions between *Orius albidipennis* (Heteroptera: Anthocoridae) and *Neoseiulus cucumeris* (Acari: Phytoseiidae): Effects of host plants under microcosm condition. Biol. Control.

[8] Weintraub P.G., Pivonia S., Steinberg S. (2011). How many *Orius laevigatus* are needed for effective western flower thrips, *Frankliniella occidentalis*, management in sweet pepper?. Crop. Prot..

[9] McMurtry J.A., Croft B.A. (1997). Life-styles of phytoseiid mites and their roles in biological control. Annu. Rev. Èntomol..

[10] Hoy M.A. (2011). Agricultural Acarology: Introduction to Integrated Mite Management.

[11] Farazmand A., Fathipour Y., Kamali K. (2015). Control of the spider mite *Tetranychus urticae* using phytoseiid and thrips predators under microcosm conditions: Single-predator versus combined-predators release. Syst. Appl. Acarol..

[12] Pakyari H. (2011). Development rate of *Scolothrips longicornis* (Thysanoptera: Thripidae) at various temperatures. Acad. J. Entomol..

[13] Osman M., Bayoumy M. (2011). Effect of prey stages of the two-spotted mite *Tetranychus urticae* on functional response of the coccinellid predator *Stethorus gilvifrons*. Acta Phytopathol. et Èntomol. Hung..

[14] Barbar Z., Kerhili S., Aslan L.H. (2016). Daily consumption and predation rate of different *Stethorus gilvifrons* (Mulsant) (Coleoptera: Coccinellidae) stages on *Panonychus citri* (McGregor) (Acari: Tetranychidae). Egypt J. Biol. Pest Control..

[15] Biddinger D.J., Weber D.C., Hull L.A. (2009). Coccinellidae as predators of mites: Stethorini in biological control. Biol. Control.

[16] Farahbakhsh G. (1961). A checklist of economically important insects and other enemies of plants and agricultural products in Iran.

[17] Kaylani S. (1967). Biology and life-history of *Stethorus gilvifrons* (Mulsant) in Lebanon. Magon Publ. Ser. Sci. Beirut.

[18] Trivedi N.P., Patel P.B., Patel J.P., Aniyaliya M.D. (2021). *Stethorus* spp.: A predator of mites. J. Pharm. Innov..

[19] Haji-Zadeh J., Kamali G.K., Assadi H.B. (1993). Investigations on the functional response and populations fluctuations of *Stethorus gilvifrons* on red spider mite, *Panonychus ulmi* (Koch) in Karaj vicinity [Iran]. J. Appl. Entomol..

[20] Taghizadeh R., Fathipour Y., Kamali K. (2008). Influence of temperature on life-table parameters of *Stethorus gilvifrons* (Mulsant) (Coleoptera: Coccinellidae) fed on *Tetranychus urticae* Koch. J. Appl. Èntomol..

[21] Taghizadeh R., Fathipour Y., Kamali K. (2008). Temperature-dependent development of acarophagous ladybird, *Stethorus gilvifrons* (Mulsant) (Coleoptera: Coccinellidae). J. Asia-Pacific Èntomol..

[22] Jafari M., Goldasteh S., Ranjbar Aghdam H., Zamani A.A., Soleyman-Nejadian E. (2019). Effect of solitary and group rearing on demographic parameters of *Stethorus gilvifrons* (Col.: Coccinelidae) feeding on *Tetranychus urticae*. J. Entomol. Res..

[23] Jafari M., Goldasteh S., Ranjbar Aghdam H., Zamani A.A., Soleyman-Nejadian E. (2020). Effect of temperature on two-sex life table parameters of *Stethorus gilvifrons* (Coleoptera: Coccinellidae) feeding on *Tetranychus urticae* (Acari: Tetranychidae). J. Entomol. Soc. Iran..

[24] Bale J.S., Masters G.J., Hodkinson I.D., Awmack C., Bezemer T.M., Brown V.K., Butterfield J., Buse A., Coulson J.C., Farrar J. (2002). Herbivory in global climate change research: Direct effects of rising temperature on insect herbivores. Glob. Chang. Biol..

[25] Dixon A.F.G. (2000). Insect Predator-Prey Dynamics: Ladybird Beetles and Biological Control.

[26] Skendžić S., Zovko M., Živković I.P., Lešić V., Lemić D. (2021). The impact of climate change on agricultural insect pests. Insects.

[27] Jerbi-Elayed M., Tougeron K., Grissa-Lebdi K., Hance T. (2022). Effect of developmental temperatures on *Aphidius colemani* host-foraging behavior at high temperature. J. Therm. Biol..

[28] Gevrey M., Worner S.P. (2006). Prediction of global distribution of insect pest species in relation to climate by using an ecological informatics method. J. Econ. Èntomol..

[29] Roy M., Brodeur J., Cloutier C. (2002). Relationship between temperature and developmental rate of *Stethorus punctillum* (Coleoptera: Coccinellidae) and its prey *Tetranychus mcdanieli* (Acarina: Tetranychidae). Environ. Èntomol..

[30] Huffaker C.B., Rabb R.L., Logan J.A., Ridgway R.L., Vinson S.B. (1977). Some aspects of population dynamics relative to augmentation of natural enemy action. Biological control by augmentation of natural enemies.

[31] Logan J., Wolesensky W., Joern A. (2006). Temperature-dependent phenology and predation in arthropod systems. Ecol. Model..

[32] Estay S.A., Lima M., Labra F.A. (2009). Predicting insect pest status under climate change scenarios: Combining experimental data and population dynamics modelling. J. Appl. Èntomol..

[33] Obrycki J.J., Tauber M.J. (1978). Thermal requirements for development of *Coleomegilla maculata* (Coleoptera: Coccinellidae) and its parasite *Perilitus coccinellae* (Hymenoptera: Braconidae). Can. Èntomol..

[34] Buckley L.B. (2022). Temperature-sensitive development shapes insect phenological responses to climate change. Curr. Opin. Insect Sci..

[35] Campbell A., Frazer B.D., Gilbert N., Gutierrez A.P., Mackauer M. (1974). Temperature requirements of some aphids and their parasites. J. Appl. Ecol..

[36] Briere J.-F., Pracros P. (1998). Comparison of temperature-dependent growth models with the development of *Lobesia botrana* (Lepidoptera: Tortricidae). Environ. Èntomol..

[37] Kontodimas D.C., Eliopoulos P.A., Stathas G.J., Economou L.P. (2004). Comparative temperature-dependent development of *Nephus includens* (Kirsch) and *Nephus bisignatus* (Boheman) (Coleoptera: Coccinellidae) preying on *Planococcus citri* (Risso) (Homoptera: Pseudococcidae): Evaluation of a linear and various nonlinear models using specific criteria. Environ. Èntomol..

[38] Aghdam H.R., Fathipour Y., Radjabi G., Rezapanah M. (2009). Temperature-dependent development and temperature thresholds of codling moth (Lepidoptera: Tortricidae) in Iran. Environ. Èntomol..

[39] Ikemoto T., Takai K. (2000). A new linearized formula for the law of total effective temperature and the evaluation of line-fitting methods with both variables subject to error. Environ. Èntomol..

[40] Analytis S. (1981). Relationship between temperature and development times in phytopathogenic fungus and in plant pests: A mathematical model. J. Agric. Res..

[41] Logan J.A., Wollkind D.J., Hoyt S.C., Tanigoshi L.K. (1976). An analytic model for description of temperature dependent rate phenomena in Arthropods. Environ. Èntomol..

[42] Lactin D.J., Holliday N.J., Johnson D.L., Craigen R. (1995). Improved rate model of temperature-dependent development by Arthropods. Environ. Èntomol..

[43] Briere J.-F., Pracros P., Le Roux A.-Y., Pierre J.-S. (1999). A novel rate model of temperature-dependent development for Arthropods. Environ. Èntomol..

[44] Analytis S. (1977). Über die Relation zwischen biologischer Entwicklung und Temperatur bei phytopathogenen Pilzen. J. Phytopathol..

[45] Analytis S. (1980). Obtaining of sub-models for modeling the entire life cycle of a pathogen/Über die Erlangung von Sub-Modellen, die zur Beschreibung eines gesamten Lebenszyklus eines Krankheitserregers dienen. Zeitschrift für Pflanzenkrankheiten und Pflanzenschutz/. J. Plant Dis. Prot..

[46] Harcourt D.G., Yee J.M. (1982). Polynomial algorithm for predicting the duration of insect life stages. Environ. Èntomol..

[47] Janisch E. (1932). The influence of temperature on the life-history of insects. Trans. R. Entomol. Soc. Lond..

[48] Taylor F. (1982). Sensitivity of physiological time in Arthropods to variation of its parameters. Environ. Èntomol..

[49] Stinner R.E., Gutierrez A.P., Butler G.D. (1974). An algorithm for temperature-dependent growth rate simulation. Can. Èntomol..

[50] Holling C.S. (1966). The functional response of invertebrate predators to prey density. Memoirs Èntomol. Soc. Can..

[51] Hilbert D.W., Logan J.A. (1983). Empirical model of nymphal development for the migratory grasshopper, *Melanoplus sanguinipes* (Orthoptera: Acrididae). Environ. Èntomol..

[52] Lamb R.J. (1992). Developmental rate of *Acyrthosiphon pisum* (Homoptera: Aphididae) at low temperatures: Implications for estimating rate parameters for insects. Environ. Èntomol..

[53] Enkegaard A. (1993). The poinsettia strain of the cotton white fly, *Bemisia tabaci* (Homoptera: Aleyrodidae), biological and demographic parameters on poinsettia (*Euphorbia pulcherrima*) in relation to temperature. Bull. Èntomol. Res..

[54] Bieri M., Baumgartner J., Bianchi G., Delucchi V., Arx R.V. (1983). Development and fecundity of pea aphid (*Acyrthosiphon pisum* Harris) as affected by constant temperatures and by pea varieties. Mitt. Schweiz. Entomol. Ges..

[55] Sharpe P.J., DeMichele D.W. (1977). Reaction kinetics of poikilotherm development. J. Theor. Biol..

[56] Akaike H. (1974). A new look at the statistical model identification. IEEE Trans. Autom. Control.

[57] Vucetich J.A., Peterson R.O., Schaefer C.C. (2002). The effect of prey and predator densities on wolf predation. Ecology.

[58] Chi H.S.I.N., Liu H. (1985). Two new methods for the study of insect population ecology. Bull. Inst. Zool. Acad. Sin..

[59] Chi H. (1988). Life-table analysis incorporating both sexes and variable development rates among individuals. Environ. Èntomol..

[60] Chi H. (2017). TWOSEX-MSChart: A computer program for the age-atage, two-sex life table analysis. http://140.120.197.173/Ecology/.

[61] Efron B., Tibshirani R.J. (1993). An Introduction to the Bootstrap.

[62] Akca I., Ayvaz T., Yazici E., Smith C.L., Chi H. (2015). Demography and population projection of *Aphis fabae* (Hemiptera: Aphididae): With additional comments on life table research criteria. J. Econ. Èntomol..

[63] Gordon R.D. (1985). The Coccinellidae (Coleoptera) of America North of Mexico. J. N. Y. Entomol..

[64] Marquardt D.W. (1963). An algorithm for least-squares estimation of nonlinear parameters. J. Soc. Ind. Appl. Math..

[65] Aksit T., Cakmak I., Ozer G. (2007). Effect of temperature and photoperiod on development and fecundity of an acarophagous ladybird beetle, *Stethorus gilvifrons*. Phytoparasitica.

[66] Shih C.I.T., Lin P.J., Chang T.W. (1991). Biology, predation, life table and intrinsic rate of increase of *Stethorus loi* Sasaji. Prot. Bull. Taipei..

[67] Fiaboe K.K.M., Gondim M.G.C., De Moraes G.J., Ogol C.K.P.O., Knapp M. (2007). Bionomics of the acarophagous ladybird beetle *Stethorus tridens* fed *Tetranychus evansi*. J. Appl. Èntomol..

[68] Khan I., Spooner-Hart R. (2017). Temperature-dependent development of immature stages of predatory ladybird beetle *Stethorus vagans* (Coleoptera: Coccinellidae) at constant and fluctuating temperatures. Acta Zoöl. Acad. Sci. Hung..

[69] Mori K., Nozawa M., Arai K., Gotoh T. (2005). Life-history traits of the acarophagous lady beetle, *Stethorus japonicus* at three constant temperatures. Biocontrol.

[70] Rattanatip J., Siri N., Chandrapatya A. (2008). Comparitive biology and lifetable of *Stethorus pauperculus* (Weise) and *S. siphonulus* (Kapur) (Coleoptera: Coccinellidae) fed on *Tetranychus urticae* Koch (Acari: Tetranychidae) in Thailand. Thai J. Agric. Sci..

[71] Perumalsamy K., Selvasundaram R., Roobakkumar A., Rahman V.J., Muraleedharan N. (2010). Life table and predatory efficiency of *Stethorus gilvifrons* (Coleoptera: Coccinellidae), an important predator of the red spider mite, *Oligonychus coffeae* (Acari: Tetranychidae), infesting tea. Exp. Appl. Acarol..

[72] Matter M.M., El-Shershaby M.M., Farag N.A., Gesraha M.A. (2011). Impact of temperature and prey density on the predacious capacity and behaviour of *Stethorus punctillum* Weise. Arch. Phytopathol. Plant Prot..

[73] Handoko H., Affandi A. (2012). Life-history traits of *Stethorus gilvifrons* (Mulsant) (Coleoptera: Coccinellidae) on phytophagous mites *Eutetranychus orientalis* Klein (Acari: Tetranychidae). AGRIVITA J. Agric. Sci..

[74] Worner S.P. (1992). Performance of phenological models under variable temperature regimes: Consequences of the Kaufmann or rate summation effect. Environ. Èntomol..

[75] Zamani A.A., Talebi A., Fathipour Y., Baniameri V. (2007). Effect of temperature on life history of *Aphidius colemani* and *Aphidius matricariae* (Hymenoptera: Braconidae), two parasitoids of *Aphis gossypii* and *Myzus persicae* (Homoptera: Aphididae). Environ. Èntomol..

[76] McCalla K.A., Keçeci M., Milosavljević I., x Ratkowsky D.A., Hoddle M.S. (2019). The influence of temperature variation on life history parameters and thermal performance curves of *Tamarixia radiata* (Hymenoptera: Eulophidae), a parasitoid of the Asian citrus psyllid (Hemiptera: Liviidae). J. Econ. Èntomol..

[77] Milosavljević I., McCalla K.A., Ratkowsky D.A., Hoddle M.S. (2019). Effects of constant and fluctuating temperatures on development rates and longevity of *Diaphorencyrtus aligarhensis* (Hymenoptera: Encyrtidae). J. Econ. Èntomol..

[78] Mutamiswa R., Tarusikirwa V., Nyamukondiwa C., Chidawanyika F. (2020). Fluctuating environments impact thermal tolerance in an invasive insect species *Bactrocera dorsalis* (Diptera: Tephritidae). J. Appl. Èntomol..

[79] Gutierrez J. (1976). Etude Biologique et Ecologique de Tetranychus neocaledonicus Andre (Acari: Tetranychidae).

[80] Bounfour M., Tanigoshi L.K. (2001). Effect of temperature on development and demographic parameters of *Tetranychus urticae* and *Eotetranychus carpini* borealis (Acari: Tetranychidae). Ann. Èntomol. Soc. Am..

[81] Praslička J., Huszár J. (2004). Influence of temperature and host plants on the development and fecundity of the spider mite *Tetranychus urticae* (Acarina: Tetranychidae). Plant Prot. Sci..

[82] El-Wahed N.A., El-Halawany A.S. (2012). Effect of temperature degrees on the biology and life table parameters of *Tetranychus urticae* Koch on two pear varieties. Egypt. Acad. J. Biol. Sci. B. Zoöl..

[83] Bayu M.S.Y.I., Ullah M.S., Takano Y., Gotoh T. (2017). Impact of constant versus fluctuating temperatures on the development and life history parameters of *Tetranychus urticae* (Acari: Tetranychidae). Exp. Appl. Acarol..

[84] Afshari M., Mossadegh S., Soleyman-Nejadian E., Kamali K. (2007). Geographical distribution and hostplants of *Stethorus gilvifrons* (Mulsant) (Col.: Coccinellidae) and its biology under laboratory conditions in Khuzestan province. J. Agri. Sci. Nat. Res..

[85] Perdikis D.C., Lykouressis D.P. (2002). Thermal requirements for development of the polyphagous predator *Macrolophus pygmaeus* (Hemiptera: Miridae). Environ. Èntomol..

[86] Lee J.-H., Ahn J.J. (2000). Temperature effects on development, fecundity, and life table parameters of *Amblyseius womersleyi* (Acari: Phytoseiidae). Environ. Èntomol..

[87] Imani Z., Shishehbor P., Sohrabi F. (2009). The effect of *Tetranychus turkestani* and *Eutetranychus orientalis* (Acari: Tetranychidae) on the development and reproduction of *Stethorus gilvifrons* (Coleoptera: Coccinellidae). J. Asia-Pacific Èntomol..

[88] Nguyen T.V., Shih C.-I.T. (2010). Development of *Neoseiulus womersleyi* (Schicha) and *Euseius ovalis* (Evans) feeding on four tetranychid mites (Acari: Phytoseiidae, Tetranychidae) and pollen. J. Asia-Pacific Èntomol..

[89] Ullah M., Gotoh T. (2014). Life-table attributes of *Neoseiulus womersleyi* (Schicha) feeding on five tetranychid mites (Acari: Phytoseiidae, Tetranychidae). Int. J. Acarol..

