# Correction: Jafari et al. Modeling Thermal Developmental Trajectories and Thermal Requirements of the Ladybird *Stethorus gilvifrons*. *Insects* 2023, *14*, 11

**DOI:** 10.3390/insects14070581

**Published:** 2023-06-26

**Authors:** Maryam Jafari, Shila Goldasteh, Hossein Ranjbar Aghdam, Abbas Ali Zamani, Ebrahim Soleyman-Nejadian, Peter Schausberger

**Affiliations:** 1 Department of Entomology, College of Agriculture, Arak Branch, Islamic Azad University, Arak 6134937333, Iran; shilagoldasteh@yahoo.com (S.G.); soley322@rocketmail.com (E.S.-N.); 2 Iranian Research Institute of Plant Protection, Agricultural Research, Education and Extension Organization, Tehran 1475744741, Iran; hrap1388@gmail.com; 3 Department of Plant Protection, College of Agriculture, Razi University, Kermanshah 6718773654, Iran; azamani@razi.ac.ir; 4 Department of Behavioral and Cognitive Biology, University of Vienna, 1030 Vienna, Austria

## Errors in Tables

Following the publication of [1], we detected some errors in Tables 1, 4 and 5. Table 1 reported an incorrect formula for the third-order polynomial model; Tables 4 and 5 contained a few incorrect numerical values. The corrected Table 1, Table 4 and Table 5 are given below; the scientific conclusions are unaffected. This correction was approved by the Academic Editor. The original publication has also been updated.

## Figures and Tables

**Table 1:** Linear and non-linear models used to simulate the developmental rates of *S. gilvifrons* at various constant temperatures.

Equation	Model	References
$D\left(T\right)=a+bT$ $T_{0}=-\frac{a}{b}\;\;\;\;K=\frac{1}{b}$	Ordinary linear regression	[35]
$DT=K+t_{min}D$	Ikemoto & Takai	[39]
$R\left(T\right)=\frac{c}{1+e^{\left(a+b.T\right)}}$	Sigmoid	[40]
$R\left(T\right)=\Psi \times \left[e^{\rho \times T}-e^{\left(\rho \times T_{max}-\frac{T_{max}-T}{\Delta T}\right)}\right]$	Logan-6	[41]
$R\left(T\right)=a\times \left[\frac{1}{1+K\times e^{-\rho \times T}}-\;e^{\left(\frac{T_{max}-T}{\Delta T}\right)\;\;}\;\right]$	Logan-10	[41]
$R\left(T\right)=e^{\rho \times T}-e^{\left(\rho \times T_{max}-\left(\frac{T_{max}-T}{\Delta T}\right)\right)}$	Lactin-1	[42]
$R\left(T\right)=e^{\rho \times T}-e^{\left(\rho \times T_{max}-\left(\frac{T_{max}-T}{\Delta T}\right)\right)+\lambda }$	Lactin-2	[42]
$R\left(T\right)=a\times T\left(T-T_{0}\right)\times \sqrt{T_{max}-T}$	Briere-1	[43]
$R\left(T\right)=a\times T\left(T-T_{0}\right)\times \sqrt[{m}]{T_{max}-T}$	Briere-2	[43]
$R\left(T\right)=a{\left(T-T_{a}\right)}^{n}\times {\left(T_{max}-T\right)}^{m}$	Analytis	[44,45]
$\;R\left(T\right)=ax^{3}+bx^{2}+cx+d$	Polynomial 3rd order	[46]
$R\left(T\right)=a{\left(T-T_{a}\right)}^{2}\left(T_{max}-T\right)$	Kontodimas-16	[37]
$D\left(T\right)=\frac{2}{D_{min}\left(e^{k\left(T-T_{opt}\right)}+e^{-\lambda \left(T-T_{opt}\right)}\right)}$	Janisch	[47]
$D\left(T\right)=R_{m}^{e\left(-as\times {\left(\frac{T-T_{m}}{T_{a}}\right)}^{a}\right)}$	Taylor	[48]
$R\left(T\right)=\frac{c}{1+e^{a+b\times T}}\;\;\;for\;\;T\;\leq T_{opt}$ $R\left(T\right)=\frac{c}{1+e^{a_{1}+b_{1}\left(2\times T_{opt\;\;\;}-T\right)}}\;for\;T>T_{opt}$	Stinner	[49]
$R\left(T\right)=\Psi \left[\frac{\left(T-T_{0}\right){\;}^{2}}{{\left(T-T_{0}\right)}^{2\;\;}+D^{2}}-e^{\left(-\frac{T_{max-\left(T-T_{0}\right)}}{\Delta }\right)}\right]$	Hilbert & Logan, or Holling III	[50,51]
$R\left(T\right)=R_{m}\times exp\left[-0.05{\left(\frac{T-T_{opt}}{T_{0}}\right)}^{2}\right]\;\;\;for\;T\leq \;T_{opt}$ $R\left(T\right)=R_{m}\times exp\left[-0.05{\left(\frac{T-T_{opt}}{T_{max}}\right)}^{2}\right]\;\;for\;T>T_{opt}$	Lamb	[52]
$\frac{1}{D}=a{\left(T-t_{min}\right)}^{2}\;\left(t_{max\;}-T\right)$	Equation-16	[37]
$\frac{1}{D}=\left(a+b\times T\right)\times \left[e\left(-\left(c+d\times T\right)\right)\right]$	Enkegaard	[53]
$\frac{1}{D}=\left[a\left(T-x_{max}\right)\right]-\left[b^{\left(T-x_{min}\right)}\right]$	Bieri-1	[54]
$\frac{1}{D}=a\left(\frac{\left(T-x_{min}\right)}{b^{\left(T-x_{min}\right)}}\right)$	Bieri-2	[54]
$R\left(T\right)=\frac{\varphi \frac{T}{T_{1}}\;exp\left[\frac{\Delta H_{A}}{R}\;\;\left(\frac{1}{T_{1}}-\frac{1}{T}\right)\right]\;\;\;\;\;\;\;\;}{1+exp\left[\frac{\Delta H_{L}}{R}\;\;\left(\frac{1}{T_{L}}-\frac{1}{T}\right)\right]+exp\left[\frac{\Delta H_{H}}{R}\left(\frac{1}{T_{H}}-\frac{1}{T}\right)\right]}$	Sharpe & DeMichele	[55]

**Table 4:** Parameter estimates for the twenty non-linear, temperature-dependent models used for describing immature development of *S. gilvifrons*.

Model	Parameters	Egg	Larva	Pupa	Total Development
Briere-1	*a*	30.51 × 10^−5^	17.50 × 10^−5^	35.61 × 10^−5^	7.86 × 10^−5^
	*t* *_min_*	13.48	14.38	13.64	13.00
	*t* *_opt_*	29.75	29.75	29.75	29.75
	*t* *_max_*	35.00	35.00	35.00	35.00
Briere-2	*a*	3.55 × 10^−4^	1.67 × 10^−6^	1.82 × 10^−4^	6.30 × 10^−9^
	*m*	3.09	0.64	1.68	0.358
	*t* *_min_*	11.17	12.20	11.24	11.96
	*t* *_opt_*	31.00	35.75	31.50	32.00
	*t* *_max_*	34.93	57.80	38.80	67.48
Logan-6	φ	−0.047	1.066	−0.045	0.570
	ρ	0.185	0.166	0.167	0.151
	Δ	6.83	5.59	6.09	6.41
	*t* *_opt_*	30.75	32.50	31.75	31.75
	*t* *_max_*	36.22	38.68	37.55	38.21
Logan-10	α	0.466	0.517	0.419	0.105
	ρ	0.236	0.176	0.315	0.291
	Δ	3.71	7.60	4.67	6.49
	*k*	249.56	138.09	847.63	586.62
	*T_L_*	38.66	44.05	44.05	44.04
	*t* *_opt_*	33.75	34.25	31.00	32.75
	*t* *_max_*	38.43	44.03	36.97	44.03
Lactin-1	Δ	5.4453	6.1696	6.0314	6.5082
	ρ	0.1834	0.1619	0.1654	0.1535
	*t_opt_*	31.00	33.00	31.09	32.00
	*t_max_*	36.3378	38.7646	37.5502	38.2170
Lactin-2	Δ	2.70	9.87	5.01	26.62
	ρ	0.01	0.011	0.018	0.017
	*e*	1.21	1.13	1.24	0.86
	*T_L_*	39.87	59.56	44.67	70.78
	*t_min_*	12.50	12.48	12.09	13.04
	*t* *_opt_*	30.75	34.00	31.50	32.25
	*t* *_max_*	36.50	47.27	36.62	46.73
Sigmoid	*a*	7.6721	5.8402	6.5996	6.2261
	*b*	−0.3775	−0.2714	−0.3224	−0.3167
	*c*	0.3105	0.1824	0.3772	0.0842
Hilbert & Logan, or Holling III	φ	0.8069	1.1954	2.6544	1.4284
	*T*_0_	5.1643	−0.0286	2.2864	−5.4646
	*d*	2.6455	34.8905	10.2500	62.5963
	*T_L_*	33.6489	71.6097	46.7278	75.7471
	Δ	17.1084	20.7993	20.0060	19.6420
	*t* *_min_*	6.18	7.3623	11.2824	12.4085
	*t* *_opt_*	31.00	36.11	31.18	33.00
	*t* *_max_*	38.21	67.99	45.91	66.11
Stinner (*T* > *T_opt_*)	*a*	−2.9618	−1.7579	−2.4292	−2.6428
	*b*	0.1898	0.1355	0.1612	0.1583
	*c*	0.3102	0.1824	0.3772	0.0842
	*t* *_opt_*	-	-	-	-
Polynomial 3rd order	*a*	1.5108	0.0316	0.9548	0.8113
	*b*	−0.2282	−0.0137	−0.1512	−0.1118
	*c*	0.0112	0.0011	0.0079	5.02 × 10^−3^
	*d*	−1.65 × 10^−4^	−1.90 × 10^−5^	−1.18 × 10^−4^	−6.97 × 10^−5^
	*t* *_opt_*	30.00	34.25	31.42	32.50
	*t* *_max_*	38.00	47.47	40.57	37.69
Equation-16	*a*	8.9669 × 10^−5^	2.2398 × 10^−5^	6.9155 × 10^−5^	1.2974 × 10^−5^
	*t* *_min_*	10.7196	8.7399	9.2019	8.0642
	*t* *_opt_*	30.00	34.00	31.40	32.00
	*t* *_max_*	39.6390	46.5227	42.4350	43.4616
Kontodimas-16	*a*	8.9669 × 10^−5^	2.2398 × 10^−5^	6.9155 × 10 ^−5^	1.2973 × 10^−5^
	*t* *_min_*	10.7196	8.7399	9.2019	8.0643
	*t* *_opt_*	30.00	34.01	31.19	31.90
	*t* *_max_*	39.6390	46.5227	42.3350	43.4615
Lamb (*T* > *T_opt_*)	*R_m_*	0.3245	0.1780	0.3782	0.0852
	*t_m_* (=*t_opt_*)	29.6197	33.1083	30.8630	31.2873
	*T*_0_	7.4244	9.7908	8.6507	9.5089
Analytis	*a*	1.3038 × 10^−15^	1.1464 × 10^−15^	1.3474 × 10^−14^	2.2536 × 10^−17^
	*n*	7.6175	6.1802	5.3244	5.8251
	*m*	2.1428	2.5713	3.9925	4.7378
	*t* *_min_*	−9.4633	−17.2948	−0.3747	−1.9163
	*t* *_opt_*	30.10	37.40	31.02	31.00
	*t* *_max_*	41.1822	60.0903	54.5616	57.4548
Enkegaard	*a*	0.0248	0.0178	0.0407	0.0122
	*b*	−68.3956 × 10^−5^	−46.1022 × 10^−5^	−10.8593 × 10^−4^	−31.9438 × 10^−5^
	*c*	1.1245	1.1075	1.1075	1.1075
	*d*	−0.1835	−0.1620	−0.1656	−0.1535
	*t* *_opt_*	31.11	33.00	31.90	32.01
	*t* *_max_*	36.66	38.76	37.55	38.21
Taylor	*R* *_m_*	0.3245	0.1780	0.3782	0.0852
	*t_m_* (=*t_opt_*)	29.6197	33.1083	30.8630	31.2874
	$T_{\sigma }$	7.4244	9.7909	8.6509	9.5093
Janisch	*D* *_min_*	3.0985	5.6899	3.6422	12.3639
	*k*	−0.1834	−0.1414	−0.2354	−0.1547
	λ	0.1221	−0.0975	−0.0364	−0.0785
	*t* *_opt_*	28.0868	30.7786	23.6969	27.90
Sharpe & DeMichele	*a*	5.1991	−10.5143	−5.2836	−8.4871
	*b*	−322.3149	−1502.5915	−259.0272	−1360.8376
	*c*	7.9817	−6.6145	−2.8555	−4.5433
	*d*	−365.7124	−1536.7211	−304.9700	−1403.3453
	*f*	17.7782	0.1576	3.076	0.1380
	*g*	−38.2689	−1276.7436	−108.8371	−1252.6541
	*t* *_opt_*	30.00	32.23	31.00	32.05
Bieri-1	*a*	0.0209	0.103	0.0249	0.0177
	*b*	1.5436	1.3103	1.274	1.0288
	*x* *_max_*	12.5366	12.4235	12.2484	−6.9928
	*x* *_min_*	37.99	45.5230	40.99	49.43
	*t* *_min_*	12.69	12.52	12.32	12.96
	*t* *_opt_*	31.01	33.50	32.00	33.04
	*t* *_max_*	36.33	41.03	39.38	49.76
Bieri-2	*a*	−0.1749	−0.0813	−0.1801	−0.0373
	*b*	0.8323	0.8504	0.8473	0.8575
	*x* *_min_*	36.33	38.76	37.55	38.21
	*t* *_opt_*	31.00	33.00	31.53	32.00
	*t* *_max_*	36.33	38.76	37.55	38.21

**Table 5:** Goodness-of-fit measures for the twenty non-linear, temperature-dependent models used for describing immature development of *S. gilvifrons*.

Model	Parameters	Egg	Larva	Pupa	Total Development
Briere-1	*R* ^2^	0.9177	0.6358	0.8053	0.7871
	*R* ^2^ *_adj_*	0.8628	0.3930	0.6755	0.6451
	*AIC*	−35.69	−35.49	−28.75	−46.31
	*RSS* (10^−4^)	57.63	83.13	183.13	72.60
Briere-2	*R* ^2^	0.9773	0.9716	0.9627	0.9923
	*R* ^2^ *_adj_*	0.9432	0.9290	0.9069	0.9807
	*AIC*	−42.72	−47.75	−38.04	−65.33
	*RSS* (10^−4^)	12.79	5.53	27.90	0.29
Logan-6	*R* ^2^	0.9634	0.9444	0.9447	0.9493
	*R* ^2^ *_adj_*	0.9085	0.8661	0.8617	0.8732
	*AIC*	−39.55	−44.16	−35.46	−54.10
	*RSS* (10^−4^)	21.70	10.05	42.90	1.92
Logan-10	*R* ^2^	0.9903	0.9757	0.9785	0.9956
	*R* ^2^ *_adj_*	0.9515	0.8785	0.8925	0.9780
	*AIC*	−47.67	−49.33	−41.12	−69.08
	*RSS* (10^−4^)	5.60	4.25	16.69	1.34
Lactin-1	*R* ^2^	0.9735	0.9696	0.9586	0.9919
	*R* ^2^ *_adj_*	0.9559	0.9494	0.9311	0.9154
	*AIC*	−43.52	−46.19	−37.45	−56.10
	*RSS* (10^−4^)	15.61	9.99	42.90	1.91
Lactin-2	*R* ^2^	0.9576	0.9648	0.9587	0.9776
	*R* ^2^ *_adj_*	0.8940	0.9120	0.8967	0.9440
	*AIC*	−31.02	−47.04	−37.41	−57.99
	*RSS* (10^−4^)	89.97	6.22	30.98	0.61
Sigmoid	*R* ^2^	0.9362	0.9777	0.9731	0.9969
	*R* ^2^ *_adj_*	0.8936	0.9629	0.9552	0.9948
	*AIC*	−38.03	−51.85	−41.50	−73.20
	*RSS* (10^−4^)	38.96	3.89	21.85	0.11
Hilbert & Logan, or Holling III	*R* ^2^	0.9792	0.9737	0.9697	0.9937
	*R* ^2^ *_adj_*	0.8963	0.8685	0.8485	0.9686
	*AIC*	−41.32	−46.84	−37.22	−64.80
	*RSS* (10^−4^)	11.57	4.61	22.91	0.23
Stinner (*T > T_opt_*)	*R* ^2^	0.9362	0.9777	0.9731	0.9969
	*R* ^2^ *_adj_*	0.8406	0.9440	0.9329	0.9949
	*AIC*	−36.03	−49.85	−39.50	−71.20
	*RSS* (10^−4^)	38.96	3.89	21.85	0.11
Polynomial 3rd order	*R* ^2^	0.9840	0.9714	0.9728	0.9924
	*R* ^2^ *_adj_*	0.9600	0.9285	0.9321	0.9810
	*AIC*	−43.44	−48.35	−39.94	−65.87
	*RSS* (10^−4^)	11.34	5.01	20.34	0.27
Equation-16	*R* ^2^	0.9696	0.9712	0.9661	0.9880
	*R* ^2^ *_adj_*	0.9493	0.9521	0.9436	0.9801
	*AIC*	−43.24	−50.31	−40.54	−65.08
	*RSS* (10^−4^)	0.16	5.03	25.63	0.42
Kontodimas-16	*R* ^2^	0.9723	0.9712	0.9659	0.9880
	*R* ^2^ *_adj_*	0.9539	0.9521	0.9433	0.9801
	*AIC*	−43.24	−50.13	−40.54	−65.08
	*RSS* (10^−4^)	16.35	5.03	25.63	0.42
Lamb (*T > T_opt_*)	*R* ^2^	0.9870	0.9674	0.9735	0.9825
	*R* ^2^ *_adj_*	0.9783	0.9456	0.9559	0.9708
	*AIC*	−35.68	−49.51	−42.08	−62.64
	*RSS* (10^−4^)	57.64	5.76	19.85	0.64
Analytis	*R* ^2^	0.9878	0.9517	0.9718	0.9848
	*R* ^2^ *_adj_*	0.9493	0.7587	0.8592	0.9241
	*AIC*	−44.37	−42.41	−37.68	−58.50
	*RSS* (10^−4^)	6.95	9.62	21.23	0.55
Enkegaard	*R* ^2^	0.9735	0.9450	0.9447	0.9492
	*R* ^2^ *_adj_*	0.9339	0.8625	0.8618	0.8732
	*AIC*	−41.52	−44.19	−35.45	−54.10
	*RSS* (10^−4^)	15.61	9.90	42.90	1.91
Taylor	*R* ^2^	0.9870	0.9674	0.9735	0.9825
	*R* ^2^ *_adj_*	0.9783	0.9456	0.9559	0.9708
	*AIC*	−47.52	−49.51	−42.08	−62.64
	*RSS* (10^−4^)	8.02	5.76	19.85	0.64
Janisch	*R* ^2^	0.9928	0.9595	0.9821	0.9743
	*R* ^2^ *_adj_*	0.9821	0.8988	0.9553	0.9358
	*AIC*	−49.55	−45.92	−42.25	−58.34
	*RSS* (10^−4^)	4.09	7.49	13.83	0.95
Sharpe & DeMichele	*R* ^2^	0.9875	0.9668	0.9759	0.9939
	*R* ^2^ *_adj_*	*	*	*	*
	*AIC*	−42.09	−45.54	−40.50	−63.08
	*RSS* (10^−4^)	7.29	5.82	18.51	0.22
Bieri-1	*R* ^2^	0.9700	0.9676	0.9565	0.9915
	*R* ^2^ *_adj_*	0.9250	0.9190	0.8913	0.9792
	*AIC*	−41.04	−47.59	−37.11	−65.24
	*RSS* (10^−4^)	16.90	5.67	32.65	0.29
Bieri-2	*R* ^2^	0.9735	0.9449	0.9447	0.9492
	*R* ^2^ *_adj_*	0.9559	0.9083	0.9079	0.9154
	*AIC*	−43.52	−49.59	−39.11	−56.10
	*RSS* (10^−4^)	15.61	9.99	42.90	1.91

* Non-calculable because the number of observations equals the number of model parameters, which results in a denominator of 0 in the equation.
